# Single-Cell Transcriptome Analysis Defines Expression of Kabuki Syndrome-Associated KMT2D Targets and Interacting Partners

**DOI:** 10.1155/2022/4969441

**Published:** 2022-08-12

**Authors:** Badam Enkhmandakh, Paul Robson, Pujan Joshi, Anushree Vijaykumar, Dong-Guk Shin, Mina Mina, Dashzeveg Bayarsaihan

**Affiliations:** ^1^Center for Regenerative Medicine and Skeletal Development, Department of Reconstructive Sciences, University of Connecticut Health Center, 263 Farmington Avenue, Farmington, CT 06030, USA; ^2^The Jackson Laboratory for Genomic Medicine, Farmington, CT 06030, USA; ^3^Computer Science and Engineering Department, University of Connecticut, 371 Fairfield Way, Unit 4155, Storrs, CT 06269, USA; ^4^Department of Craniofacial Sciences, University of Connecticut Health Center, 263 Farmington Avenue, Farmington, CT 06030, USA; ^5^Institute for System Genomics, University of Connecticut, Engineering Science Building Rm. 305, 67 North Eagleville Road, Storrs, CT 06269, USA

## Abstract

*Objectives*. Kabuki syndrome (KS) is a rare genetic disorder characterized by developmental delay, retarded growth, and cardiac, gastrointestinal, neurocognitive, renal, craniofacial, dental, and skeletal defects. KS is caused by mutations in the genes encoding histone H3 lysine 4 methyltransferase (KMT2D) and histone H3 lysine 27 demethylase (KDM6A), which are core components of the complex of proteins associated with histone H3 lysine 4 methyltransferase SET1 (SET1/COMPASS). Using single-cell RNA data, we examined the expression profiles of *Kmt2d* and *Kdm6a* in the mouse dental pulp. In the incisor pulp, *Kmt2d* and *Kdm6a* colocalize with other genes of the SET1/COMPASS complex comprised of the WD-repeat protein 5 gene (*Wdr5*), the retinoblastoma-binding protein 5 gene (*Rbbp5*), absent, small, and homeotic 2-like protein-encoding gene (*Ash2l*), nuclear receptor cofactor 6 gene (*Ncoa6*), and Pax-interacting protein 1 gene (*Ptip1*). In addition, we found that *Kmt2d* and *Kdm6a* coexpress with the downstream target genes of the Wingless and Integrated (WNT) and sonic hedgehog signaling pathways in mesenchymal stem/stromal cells (MSCs) at different stages of osteogenic differentiation. Taken together, our results suggest an essential role of KMT2D and KDK6A in directing lineage-specific gene expression during differentiation of MSCs.

## 1. Introduction

Kabuki syndrome (KS) is a rare genetic disorder caused by mutations in the histone modifier genes encoding histone H3 lysine 4 methyltransferase (*KMT2D*) and histone H3 lysine 27 demethylase (*KDM6A*) (OMIM: #147920 and #300867) [1, 2]. The main clinical manifestations of KS include dysmorphic facial features, skeletal abnormalities, intellectual disability, hearing loss, and retarded postnatal growth. In addition, KS frequently associates with various dental abnormalities such as abnormal tooth number, hypodontia, microdontia, widely spaced teeth, and enamel hypoplasia [3–5].

Mutations in *KMT2D* are the most common cause of KS and account for 75% of cases, whereas mutations in *KDM6A* cause up to 5% of cases [6–8]. In mice, *Kmt2d* and *Kdm6a* are essential during early embryonic development and exhibit a broad and distinct expression pattern in most adult tissues [6, 7]. As part of the complex of proteins associated with histone H3 lysine 4 methyltransferase SET1 (SET1/COMPASS), KMT2D and KDM6A physically associate with a protein module comprised of the WD-repeat protein 5 (WDR5), retinoblastoma-binding protein 5 (RBBP5), absent, small, and homeotic 2-like protein-encoding protein (ASH2L) and Dpy-30 histone methyltransferase complex regulatory subunit (DPY30) and nuclear receptor cofactor 6 (NCOA6), Pax-interacting protein 1 (PTIP), and PTIP-associated protein 1 (PA1) [6]. Recent research has shown that association of KMT2D with the histone acetyltransferases p300 and CBP encoded by *EP300* and *CREBBP* is capable of establishing active enhancer states enriched in histone H3 lysine 4 monomethylation and histone H3 lysine 27 acetylation (H3K4me1/H3K27ac) to facilitate long-distance gene activation [9]. In addition to p300/CBP, the interplay between KMT2D and the SWI/SNF related, matrix associated, and actin-dependent ATP-dependent chromatin remodeling factors (SMARCA4 and SMARCB1) promotes cell type-specific enhancer activation [10]. Research has demonstrated that haploinsufficiency of KMT2D is sufficient to lead to the classical KS phenotype [11]. Mechanistically, haploinsufficiency of KMT2D causes structural changes in chromatin, which affects the mechanical properties of the nucleus [12]. By contrast, haploinsufficiency of *KDM6A* leads to postnatal growth restriction, microcephaly, cerebral atrophy, seizures, facial dysmorphism, and cleft palate [13]. KDM6A plays an important role in definitive endoderm differentiation through modulating the Wingless and Integrated (WNT) signaling pathway [14]. A recent study also established that KDM6A controls human neural differentiation and dendritic morphology [15]. KDM6A is capable of changing the composition of bivalent promoters by removing histone H3 lysine 27 trimethylation (H3K27me3) marks, which in turn leads to selective upregulation of neural genes. These findings are also supported by work by Dhar et al. showing that KDM6A is required for the activation of bivalent genes during mouse embryonic stem cell differentiation [16].

Previously, using single-cell RNA sequencing (scRNA-seq), we characterized the cellular composition of the mouse incisor dental pulp [17]. Our study revealed distinct patterns of cell state heterogeneity in mesenchymal stem/stromal cells (MSCs) undergoing different stages of differentiation, including differentiated cells representing osteoblasts and odontoblasts. In this study, we examined the expression profile of *Kmt2d* and *Kdm6a* in different subpopulations of pulp cells. Our investigation revealed that genes encoding members of the WRAD protein complex display partially overlapping expression patterns in MSCs. We also noted that members of the WNT and sonic hedgehog signaling (SHH) pathways, which are known downstream targets of KMT2D [18], exhibit similar expression patterns across distinct subclasses of pulp cells. Collectively, our analysis sheds new light on the role of KMT2D and KDK6A in the lineage commitment of MSCs.

## 2. Materials and Methods

### 2.1. scRNA-seq

Incisor dental pulps from 6-day wild-type mice were obtained as described previously [19]. The cell suspension was then assessed with a Countess II FL Automated Cell Counter (Thermo Fisher Scientific Inc., Waltham, MA). A total of 8,000 cells were loaded into for capture into single channels of the 10× Genomics Chromium Controller (10× Genomics, Pleasanton, CA). After cell lysis, complementary DNA was synthesized and amplified for library preparation and sequencing with the HiSeq 4000 (Illumina, San Diego, CA).

### 2.2. Genome-Wide ATAC-seq, hMeDIP-seq, and Bulk RNA-seq

We used the Active Motif (Carlsbad, CA) service to perform Assay for Transposase-Accessible Chromatin using sequencing (ATAC-seq), hydroxymethylated DNA immunoprecipitation sequencing (hMeDIP-seq), and bulk RNA-seq. The extraction of RNA was performed with the RNAeasy Mini/Midi kit (Qiagen, Germantown, MD). Whole-transcriptome analysis was performed with the Illumina NextSeq 500. The Burrows-Wheeler Aligner (BWA) algorithm with default settings was used to map the paired-end sequencing reads to the mouse genome. For the hMeDIP-seq experiment, we applied the Monarch Genomic DNA Isolation kit (New England Biolabs, Ipswich, MA) to isolate genomic DNA. The sonicated DNA was then ligated to the Illumina adaptors. The antibody AM39791 to 5hmC was used to produce DNA demethylation tags. Next, the libraries were generated from immunoprecipitated DNA and sequenced with the NextSeq 500. Input DNA without the immunoprecipitation step was used as a control.

### 2.3. Computational Analysis

The sequencing reads with more than one mismatch were excluded. The STAR aligner was used, and only reads with MAPQ scores greater than 255 were included. The 10× Genomics barcodes and a unique molecular identifier threshold were used for filtering and generation of a pulp digital counts matrix. The expression pattern of the pulp cells was measured by dispersion and dimensionality reduction with uniform manifold approximation and projection (UMAP) [20–22]. The neighborhood clustering graph was performed with the Leiden algorithm [23]. The neighborhood graph was corrected using the batch remover BBKNN [24]. The Illumina base call files were converted to FASTQ format and aligned to the mm10 genome. The MACS2 peak-calling program was used for determining chromatin accessibility across the genome [25]. The BWA algorithm with default settings was used to map hMeDIP-seq reads. The MACS peak finding algorithm was used to map methylated segments (18). The 5hmC enrichment was presented as average of values for all target regions. The STAR aligner and the Subread package were used for RNA-seq fragments, feature counts (FPKM assignment to genes), and DESeq2 differential analysis [26–28].

## 3. Results

### 3.1. Expression of Members of the SET1/COMPASS and PRC2 Complexes Associated with KMT2D and KDM6A

**Figure 1:**
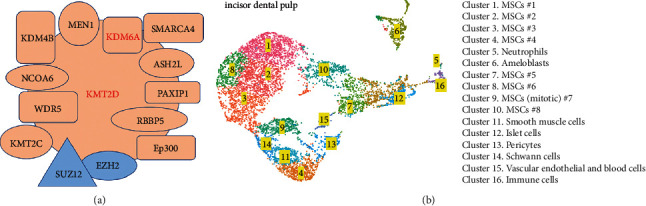
The interaction network of KMT2D and KDM6A and scRNA-seq clustering analysis of the mouse incisor dental pulp. (a) STRING interaction network analysis revealed that both KMT2D and KDM6A associate with other components of the SET1/COMPASS complex, including KMT2C, ASH2L, WDR5, RBBP5, KDM4B, p300, NCOA6, SMARCA4, PAXIP1, and MEN1. In addition, this analysis revealed that KMT2D and KDM6A physically interact with EZH2 and SUZ12, key components of the PRC2. (b) The mouse incisor dental pulp is composed of 16 distinct cell types that represent MSCs at different stages of osteogenic and odontogenic differentiation as well as pericytes, ameloblasts, smooth muscle cells, islet cells, Schwann cells, vascular endothelial, and blood cells.

**Figure 2:**
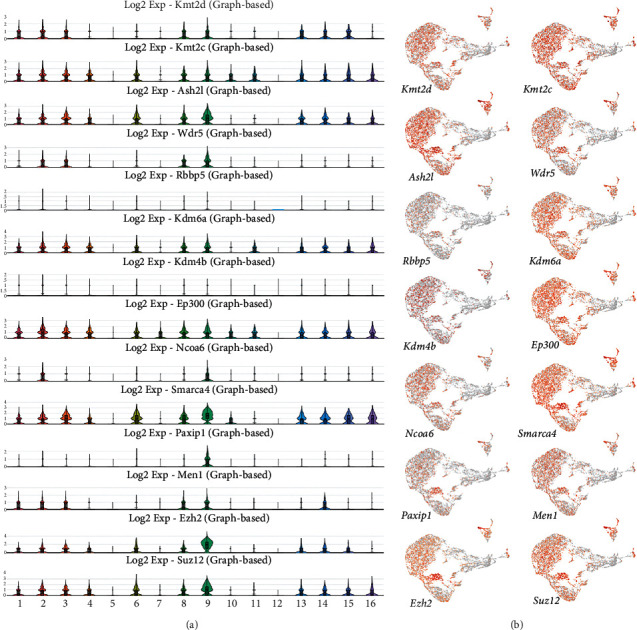
Expression of genes encoding proteins associated with KMT2D and KDM6A. (a) Violin plots of genes encoding KMT2C, ASH2L, WDR5, RBBP5, KDM4B, p300, NCOA6, SMARCA4, PAXIP1, MEN1, EZH2, and SUZ12. (b) UMAP visualization of genes encoding components of the SET1/COMPASS and PRC2 complexes.

KMT2D and KDM6A function as components of the transcriptional activation complex SET1/COMPASS to establish open chromatin domains associated with active enhancers (H3K4me1-rich) and promoters (H3K4me2/3-rich) [6]. Protein-protein interaction networks are critical for a system-level understanding of gene regulatory processes. By analyzing the Search Tool for the Retrieval of Interacting Genes/Proteins (STRING) interaction network (https://string-db.org), we discovered that both KMT2D and KDM6A have a specific set of interacting partners (Figure 1(a)). Among the members of the SET1/COMPASS complex, we identified KMT2C, ASH2L, WDR5, RBBP5, KDM4B, p300, NCOA6, SMARCA4, PAXIP1, and MEN1. The STRING analysis revealed that enhancer of zeste homolog 2 (EZH2) and suppressor of zeste 12 protein homolog (SUZ12), key components of the polycomb repressive complex 2 (PRC2), physically interact with KMT2D and KDM6A. Previously, we reported the results of an scRNA-seq analysis to define the expression pattern of developmental genes in the incisor dental pulp [17]. Based on the expression of key genes, we grouped the pulp cells into 16 clusters (Figure 1(b)). In the current study, we performed a more in-depth analysis of the *Kmt2d* and *Kdm6a* expression. *Kmt2d* is mainly expressed in clusters 1, 2, 3 8, 9, 13, 14, and 15, whereas *Kdm6a* displayed a broader expression domain with enrichment in clusters 1, 2, 3, 4, 6, 8, 9, 13, 14, 15, and 16 (Figure 2). Additionally, we analyzed the expression of genes encoding critical partners of KMT2D and KDM6A within the SET1/COMPASS complex. Similar to the expression pattern of *Kdm6a*, *Ash2l* is enriched in clusters 1, 2, 3, 4, 6, 8, 9, 13, 14, 15, and 16. *Wdr5* is expressed in clusters 2, 3, 8, and 9, while *Rbbp2* and lysine demethylase 4b gene (*Kdm4b)* are relatively weakly expressed in dental pulp. *Ep300* is vigorously expressed in clusters 1, 2, 3, 4, 6, 7, 8, 9, 10, 11, 13, 14, 15, and 16. *Ncoa6* has limited expression and is mainly enriched in clusters 2 and 9. *Smarca4* exhibits a wide expression range in clusters 1, 2, 3, 4, 6, 8, 9, 10, 13, 14, 15, and 16. The expression of *Paxip1* is limited to cluster 9, whereas *Men1* is enriched in clusters 1, 2, 3, 8, 9, and 14. In addition to the members of the SET1/COMPASS complex, we analyzed the expression profiles of genes encoding EZH2 and SUZ12. *Ezh2* is enriched in clusters 1, 2, 3, 4, 6, 8, 9, 13, 14, and 15. *Suz12* displays a very similar expression profile with enrichment in clusters 1, 2, 3, 4, 6, 8, 9, 13, 14, 15, and 16 (Figure 2).

### 3.2. Expression of Downstream Targets of KMT2D Associated with the WNT and SHH Signaling Pathways

**Figure 3:**
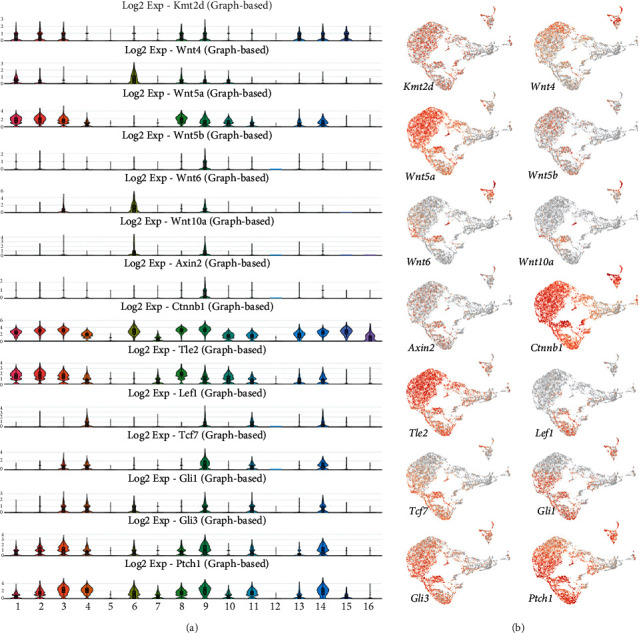
Expression of genes encoding downstream targets of KMT2D and KDM6A. (a) Violin plots of genes encoding *Wnt5a*, *Ctnnb1*, *Tle*2, *Gli3*, and *Ptch1*. (b) UMAP visualization of genes encoding components of the WNT and SHH signaling pathways.

We analyzed the expression profiles of members of the WNT and SHH signaling that are known to be downstream targets of KMT2D. We detected expression of *Wnt4* in clusters 1, 2, 6, 8, 9, and 10 (Figure 3). By contrast, *Wnt5a* is more vigorously expressed in clusters 1, 2, 3, 4, 8, 9, 10, 11, 13, and 14, and *Wnt5b* expression is limited to cluster 9. *Wnt6* is enriched in clusters 3, 6, and 9. The expression of *Wnt10a* is restricted to clusters 6 and 9. *Axin2* encoding axis inhibition protein 2 is only enriched in cluster 9. The *Ctnnb1* gene encoding b-catenin displayed very broad and vigorous expression in the incisor dental pulp, with enrichment in clusters 1, 2, 3, 4, 6, 7, 8, 9, 10, 11, 13, 14, 15, and 16. *Tle2* encodes transducing-like protein 2, which acts as a transcriptional repressor [29]. We identified high expression of *Tle2* in clusters 1, 2, 3, 4, 7, 8, 9, 10, 11, 13, and 14. *Lef1* and *Tcf7*, which encode transcription factors of the WNT pathway, showed a relatively restricted range of expression; *Lef1* is detected in clusters 4, 9, and 11, whereas *Tcf7* is only enriched in clusters 3, 4, 9, 11, and 14. We also analyzed the expression patterns of members of SHH signaling such as *Gli1*, *Gli3*, and *Ptch1*. *Gli1* exhibits predominant expression in clusters 3, 4, 6, 9, 11, and 14. *Gli3* has a broad expression pattern with a relatively high expression in clusters 1, 2, 3, 4, 6, 8, 9, 11, 13, and 14. *Ptch1* is also broadly expressed, enriched in clusters 1, 2, 3, 4, 6, 7, 8, 9, 10, 11, 13, 14, and 15 (Figure 3).

### 3.3. Chromatin Accessibility of the KMT2D- and KDM6-Associated Factors and Downstream Target Genes

**Figure 4:**
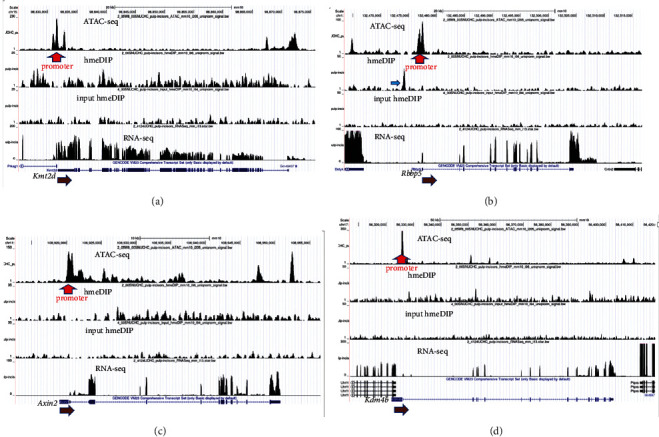
Chromatin structures of *Kmt2d*, *Rbbp5*, *Axin2*, and *Kdm4b* in the mouse incisor dental pulp. (a) ATAC-seq analysis indicated that the chromatin is accessible in *Kmt2d.* Strong peaks were detected in the transcription start site (TSS) marked with a red arrow. hmeDIP-seq results showed 5hmC enrichment in gene bodies (compare to the input), which correlates well with RNA expression. The brown arrow indicates the direction of transcription. (b) The hmeDIP-seq and ATAC-seq peaks are enriched in the promoter (blue arrow) and TSS (red arrow) of *Rbbp5*. The brown arrow indicates the direction of *Rbbp5* expression. (c) The ATAC-seq and hmeDIP-seq peaks are enriched in the gene body of *Axin2*. A strong ATAC-seq signal was also detected in the TSS (red arrow). The direction of *Axin2* expression is marked with a brown arrow. (d) A strong ATAC-seq peak is present in the TSS (red arrow) of *Kdm4b*. 5hmC is enriched within the gene body of *Kdm4b*. The brown arrow indicates the direction of *Kdm4b* expression.

According to the scRNA-seq data, *Rbbp3*, *Kdm4b*, and *Axin2* exhibit relatively weak expression in the dental pulp (Figures 2 and 3). We next investigated the genomic structure of these genes for specific epigenetic marks and chromatin accessibility. Open chromatin regions and the DNA demethylation mark 5hmC are reliable indicators of active genomic states. Previously, using ATAC-seq and hmeDIP-seq assays, we identified accessible chromatin regions and genome-wide enrichment of 5hmC in the mouse dental pulp [30, 31]. By analyzing these datasets, we found that the vast majority of genes encoding protein partners and downstream targets of KMT2D and KDM6A exhibit open chromatin enriched in 5hmC (data not shown). Our analysis also revealed that, despite the weak expression of *Rbbp3*, *Kdm4b*, and *Axin2*, the genomic regions across these genes retain open chromatin configurations (Figure 4). Additionally, we detected specific enrichment of 5hmC in *Rbbp3*, *Kdm4b*, and *Axin2*. Collectively, these data suggest that even relatively weakly expressed genes acquire active chromatin states in the mouse dental pulp.

## 4. Discussion

**Figure 5:**
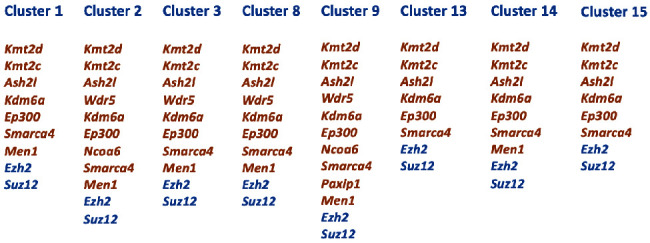
Core set of epigenetic factors that are common in five subpopulations of MSCs, pericytes, Schwann cells, and vascular endothelial cells. These factors are KMT2D, KDM6A, KMT2C, ASH2L, p300, and SMARCA4, which are the key components of the SET1/COMPASS complex (brown), as well as EZH2 and SUZ12 (blue), which are subunits of the PRC2.

**Figure 6:**
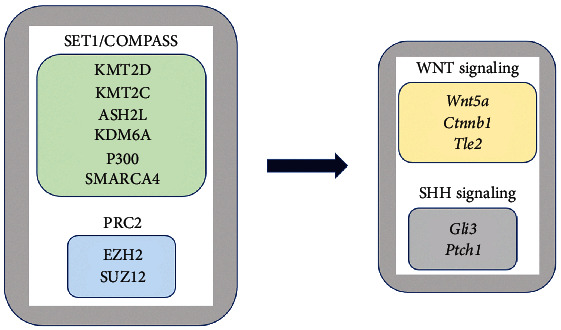
KMT2D and KDM6A form functional interactions with other components of the SET1/COMPASS complex and the PRC2 to control the expression of downstream targets of the WNT and SHH signaling pathways.

Our study revealed that genes encoding KMT2D and KDM6A and other components of the SET1/COMPASS activation complex display overlapping expression patterns within the mouse dental pulp. Sixteen clusters of cells represent MSCs at different stages of osteogenic and odontogenic differentiation as well as pericytes, ameloblasts, smooth muscle cells, islet cells, Schwann cells, vascular endothelial cells, and blood cells [17]. We identified a core set of genes that are common in clusters 1, 2, 3, 8, and 9, which represent the five subpopulations of MSCs (Figure 5). These genes encode KMT2D, KDM6A, KMT2C, ASH2L, p300, and SMARCA4, which are key components of the SET1/COMPASS complex (Figure 6). The mouse STRING database revealed that KMT2D and KDM6A also interact with EZH2 and SUZ12, which are subunits of the PRC2. We analyzed the expression patterns of *Ezh2* and *Suz12* in the incisor dental pulp and found that both genes exhibit vigorous expression in all pulp clusters including MSCs.

**Figure 7:**
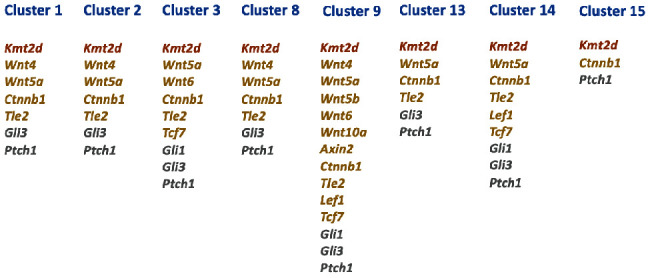
Core set of genes that are common in five subpopulations of MSCs. These genes are *Wnt5a*, *Ctnnb1*, *Tle2*, *Gli3*, and *Ptch1*. *Kmt2d* is marked in brown. Members of the WNT signaling pathway are shown in light brown and members of SHH signaling are in grey.

A recent investigation reported the involvement of KMT2D in regulating WNT/*β*-catenin and SHH signaling in dental epithelium [18]. Both *KMT2D* and *KDM6A* are coexpressed in the dental epithelium of human tooth germs [32]. Hence, we examined the expression profiles of the canonical targets of KMT2D in the dental pulp. We deduced that *Wnt5a*, *Ctnnb1*, *Tle*2, *Gli3*, and *Ptch1* represent a common set of genes for all five clusters of MSCs (Figures 6 and 7).

KMT2D is a major mammalian histone H3K4 methyltransferase that interacts with transcription factors and chromatin remodeling proteins to mediate transcriptional activation [33]. Specific nonsense and frameshift mutations in the *KMT2D* sequence have been reported to lead to KS, which is characterized by a dysmorphic face, postnatal growth retardation, skeletal abnormalities, midfacial hypoplasia, cleft lip/palate, and mental problems [34, 35]. Common dental abnormalities in KS patients are ectopic upper molars, screwdriver-shaped upper incisors, delayed tooth eruption, widely spaced teeth, enamel hypoplasia, missing teeth, high-arched palate, micrognathia, small dental arches, hypodontia, microdontia, severe maxillary recession, congenital absence of teeth, and malocclusion [4, 5, 35–38].

With the assistance of histone H3K27 acetyltransferases CBP and p300, KMT2D is involved in enhancer activation and cell-type-specific gene expression during differentiation [33]. In chondrocytes, KMT2D regulates the expression of *Shox2* [39]. Research has established that a decrease in *Shox2* expression in *Kmt2d*-depleted mouse chondrocytes can release *Sox9* inhibition, thereby causing chondrocyte differentiation. *Kmt2d* is expressed in the developing mouse calvarial osteoblasts, epithelia, and neural tissues [40]. Moreover, the heterozygous loss of *Kmt2d* impairs the neuromuscular junction, muscle cell differentiation, and myofiber regeneration [2]. In addition, a growing body of research indicates that defects in neural crest development are a major cause of KS [41, 42]. Mouse knockout studies have revealed that *Kmt2d* and *Kdm6a* are required for proper differentiation of cranial neural crest cells [42, 43]. *Kmt2d* depletion in *Xenopus* impairs neural crest formation, which is accompanied by reduced levels of H3K4me1 and H3K27ac, a hallmark feature of active chromatin states [41]. Interestingly, in *Danio*, inactivation of *Kmt2d* affected all examined tissues, whereas ablation of *Kdm6a* had a more selective impact on craniofacial and heart development [44]. These findings provide mechanistic insights into the pathogenesis of KS and indicate KMT2D as a key regulator of development and differentiation.

The dental pulp-derived MSCs exhibit multipotent differentiation capacity and are considered a promising stem cell source for tissue engineering and regenerative medicine [45]. Over the past decades, a growing body of scientific evidence shows that epigenetic mechanisms play a major role in lineage specification and gene regulatory networks underlying MSC differentiation [46–48]. Therefore, further research toward identifying the molecular pathways through which KMT2D controls gene expression in dental pulp stem cells will provide a more refined understanding of the mechanisms underlying the pathogenesis of KS.

## Data Availability

All datasets from this study will be made available upon request.

## References

[1] Aristizábal E., Diaz-Ordóñez L., Candelo E., Pachajoa H. (2021). A novel Intronic KMT2D variant as a cause of kabuki syndrome: a case report. *Applied Clinical Genetics*.

[2] Wright A., Hall A., Daly T. (2021). Lysine methyltransferase 2D regulates muscle fiber size and muscle cell differentiation. *FASEB Journal*.

[3] Sobral D. S., Leite A. F., Figueiredo P. T. (2013). Craniofacial and dental features in Kabuki syndrome patients. *Cleft Palate and Craniofacial Journal*.

[4] Tuna E. B., Marşan G., Gençay K., Seymen F. (2012). Craniofacial and dental characteristics of kabuki syndrome: nine years cephalometric follow-up. *Journal of Clinical and Pediatric Dentistry*.

[5] Silva-Andrade N., López-Ortega K., Gallottini M. (2019). Orofacial features and medical profile of eight individuals with Kabukis yndrome. *Medicina Oral Patologia Oral Cirugia Bucal*.

[6] Froimchuk E., Jang Y., Ge K. (2017). Histone H3 lysine 4 methyltransferase KMT2D. *Gene*.

[7] Tran N., Broun A., Ge K. (2020). Lysine demethylase KDM6A in differentiation, development, and cancer. *Molecular and Cellular Biology*.

[8] Boniel S., Szymańska K., Śmigiel R., Szczałuba K. (2021). Kabuki syndrome-clinical review with molecular aspects. *Genes (Basel)*.

[9] Wang L. H., Aberin M. A. E., Wu S., Wang S. P. (2021). The MLL3/4 H3K4 methyltransferase complex in establishing an active enhancer landscape. *Biochemical Society Transactions*.

[10] Park Y. K., Lee J. E., Yan Z. (2021). Interplay of BAF and MLL4 promotes cell type-specific enhancer activation. *Nature Communications*.

[11] Luperchio T. R., Applegate C. D., Bodamer O., Bjornsson H. T. (2020). Haploinsufficiency of KMT2D is sufficient to cause Kabuki syndrome and is compatible with life. *Molecular Genetics and Genomic Medicine*.

[12] Fasciani A., D’Annunzio S., Poli V. (2020). MLL4-associated condensates counterbalance polycomb-mediated nuclear mechanical stress in Kabuki syndrome. *Nature Genetics*.

[13] Lindgren A. M., Hoyos T., Talkowski M. E. (2013). Haploinsufficiency of KDM6A is associated with severe psychomotor retardation, global growth restriction, seizures and cleft palate. *Human Genetics*.

[14] Jiang W., Wang J., Zhang Y. (2013). Histone H3K27me3 demethylases KDM6A and KDM6B modulate definitive endoderm differentiation from human ESCs by regulating WNT signaling pathway. *Cell Research*.

[15] Tang Q. Y., Zhang S. F., Dai S. K. (2020). UTX regulates human neural differentiation and dendritic morphology by resolving bivalent promoters. *Stem Cell Reports*.

[16] Dhar S. S., Lee S. H., Chen K. (2016). An essential role for UTX in resolution and activation of bivalent promoters. *Nucleic Acids Research*.

[17] Bayarsaihan D., Enkhmandakh B., Vijaykumar A., Robson P., Mina M. (2021). Single-cell transcriptome analysis defines mesenchymal stromal cells in the mouse incisor dental pulp. *Gene Expression Patterns*.

[18] Pang L., Tian H., Gao X., Wang W., Wang X., Zhang Z. (2021). KMT2D deficiency disturbs the proliferation and cell cycle activity of dental epithelial cell line (LS8) partially via Wnt signaling. *Bioscience Reports*.

[19] Balic A., Aguila H. L., Caimano M. J., Francone V. P., Mina M. (2010). Characterization of stem and progenitor cells in the dental pulp of erupted and unerupted murine molars. *Bone*.

[20] Satija R., Farrell J. A., Gennert D., Schier A. F., Regev A. (2015). Spatial reconstruction of single-cell gene expression data. *Nature Biotechnology*.

[21] Zheng G. X., Terry J. M., Belgrader P. (2017). Massively parallel digital transcriptional profiling of single cells. *Nature Communications*.

[22] Becht E., McInnes L., Healy J. (2019). Dimensionality reduction for visualizing single-cell data using UMAP. *Nature Biotechnology*.

[23] Traag V. A., Waltman L., van Eck N. J. (2019). From Louvain to Leiden: guaranteeing well-connected communities. *Science Reports*.

[24] Polański K., Young M. D., Miao Z., Meyer K. B., Teichmann S. A., Park J. E. (2020). BBKNN: fast batch alignment of single cell transcriptomes. *Bioinformatics*.

[25] Zhang Y., Liu T., Meyer C. A. (2008). Model-based analysis of ChIP-Seq (MACS). *Genome Biology*.

[26] Dobin A., Davis C. A., Schlesinger F. (2013). STAR: ultrafast universal RNA-seq aligner. *Bioinformatics*.

[27] Liao Y., Smyth G. K., Shi W. (2014). FeatureCounts: an efficient general-purpose program for assigning sequence reads to genomic features. *Bioinformatics*.

[28] Love M. L., Huber W., Anders S. M. (2014). Moderated estimation of fold change and dispersion for RNA-seq data with DESeq2. *Genome Biology*.

[29] Grbavec D., Lo R., Liu Y., Stifani S. (1998). Transducin-like enhancer of split 2, a mammalian homologue of Drosophila Groucho, acts as a transcriptional repressor, interacts with hairy/enhancer of split proteins, and is expressed during neuronal development. *European Journal of Biochemistry*.

[30] Joshi P., Vijaykumar A., Enkhmandakh B., Mina M., Shin D. G., Bayarsaihan D. (2022). Genome-wide distribution of 5hmC in the dental pulp of mouse molars and incisors. *Journal of Biochemistry*.

[31] Joshi P., Vijaykumar A., Enkhmandakh B., Shin D. G., Mina M., Bayarsaihan D. (2022). The chromatin accessibility landscape in the dental pulp of mouse molars and incisors. *Acta Biochimica Polonica*.

[32] Porntaveetus T., Abid M. F., Theerapanon T. (2018). Expanding the oro-dental and mutational spectra of Kabuki syndrome and expression of KMT2D and KDM6A in human tooth germs. *International Journal of Biological Sciences*.

[33] Bayarsaihan D. (2018). Modus operandi of COMPASS/MLL epigenetic writers in the mammalian genome. *Epigenomics*.

[34] Cogulu D., Oncag O., Celen E., Ozkinay F. (2008). Kabuki syndrome with additional dental findings: a case report. *Journal of Dentistry for Children*.

[35] Teixeira C. S., Silva C. R., Honjo R. S., Bertola D. R., Albano L. M. J., Kim C. A. (2009). Dental evaluation of Kabuki syndrome patients. *Cleft Palate and Craniofacial Journal*.

[36] Mhanni A. A., Cross H. G., Chudley A. E. (1999). Kabuki syndrome: description of dental findings in 8 patients. *Clinical Genetics*.

[37] Matsune K., Shimizu T., Tohma T., Asada Y., Ohashi H., Maeda T. (2001). Craniofacial and dental characteristics of Kabuki syndrome. *American Journal of Medical Genetics*.

[38] Petzold D., Kratzsch E., Opitz C., Tinschert S. (2003). The Kabuki syndrome: four patients with oral abnormalities. *European Journal of Orthodontics*.

[39] Fahrner J. A., Lin W. Y., Riddle R. C. (2019). Precocious chondrocyte differentiation disrupts skeletal growth in Kabuki syndrome mice. *Journal of Clinical Investigation Insight*.

[40] Dong C., Umar M., Bartoletti G., Gahankari A., Fidelak L., He F. (2019). Expression pattern of Kmt2d in murine craniofacial tissues. *Gene Expression Patterns*.

[41] Schwenty-Lara J., Nehl D., Borchers A. (2020). The histone methyltransferase KMT2D, mutated in Kabuki syndrome patients, is required for neural crest cell formation and migration. *Human Molecular Genetics*.

[42] Shpargel K. B., Mangini C. L., Xie G., Ge K., Magnuson T. (2020). The KMT2D Kabuki syndrome histone methylase controls neural crest cell differentiation and facial morphology. *Development*.

[43] Shpargel K. B., Starmer J., Wang C., Ge K., Magnuson T. (2017). UTX-guided neural crest function underlies craniofacial features of Kabuki syndrome. *Proceedings of the National Academy of Sciences of the United States of America*.

[44] Van Laarhoven P. M., Neitzel L. R., Quintana A. M. (2015). Kabuki syndrome genes KMT2D and KDM6A: functional analyses demonstrate critical roles in craniofacial, heart and brain development. *Human Molecular Genetics*.

[45] Liu Y., Gan L., Cui D. X. (2021). Epigenetic regulation of dental pulp stem cells and its potential in regenerative endodontics. *World Journal of Stem Cells*.

[46] Bayarsaihan D. (2016). Deciphering the epigenetic code in embryonic and dental pulp stem cells. *The Yale Journal of Biology and Medicine*.

[47] Zhou D., Gan L., Peng Y. (2020). Epigenetic regulation of dental pulp stem cell fate. *Stem Cells International*.

[48] Zhang Q., Huang Z., Zuo H. (2021). Chromatin accessibility predetermines odontoblast terminal differentiation. *Frontiers in Cell and Developmental Biology*.

